# Mind the gap: Adherence to denosumab dosing and cessation guidelines in Australian residential aged care

**DOI:** 10.1002/bcp.70574

**Published:** 2026-04-23

**Authors:** Marea O'Donnell, Johanna I. Westbrook, Bayzidur Rahman, Isabelle Meulenbroeks, Nasir Wabe, Ian D. Cameron, Mark Morgan, Magdalena Z. Raban

**Affiliations:** ^1^ Centre for Health Systems and Safety Research, Australian Institute of Health Innovation Macquarie University Sydney New South Wales Australia; ^2^ John Walsh Centre for Rehabilitation Research Northern Sydney Local Health District Sydney New South Wales Australia; ^3^ Faculty of Medicine and Health The University of Sydney Sydney New South Wales Australia; ^4^ Faculty of Health Sciences and Medicine Bond University Gold Coast Queensland Australia

**Keywords:** adherence, aged care, denosumab, medication administration, osteoporosis

## Abstract

**Aims:**

Denosumab and bisphosphonates are the main treatments for osteoporosis in residential aged care (RAC). Strict adherence to 6‐monthly denosumab dosing is critical as delayed doses can result in bone resorption and vertebral fractures. If denosumab is ceased, then a bisphosphonate is recommended to avoid vertebral fractures. We aimed to examine adherence to denosumab dosing guidelines, practices of ceasing or switching to bisphosphonate treatment and how these vary in Australian RAC.

**Methods:**

A retrospective cohort study using routinely collected electronic medication administration data. Between 2018 and 2022, a total of 10 674 residents were treated for osteoporosis with denosumab or bisphosphates in 413 RAC homes. Non‐adherence to denosumab treatment guidelines was defined as either a dosing interval of >210 days or cessation of denosumab without bisphosphonate replacement (residents with ≥12‐month follow‐up). Secondary outcomes were residents who ceased or switched from a bisphosphonate.

**Results:**

A total of 9281 residents (86.9%) were administered denosumab, and 5986 were administered more than one dose. In total, there were 15 040 intervals between consecutive denosumab doses, and 14.8% (*n* = 2222) were longer than recommended by guidelines, affecting 20.0% (*n* = 1881) of residents on denosumab. Of residents who ceased denosumab, 98.2% (*n* = 833) were not administered a bisphosphonate. Of residents who ceased a bisphosphonate, 58.9% (*n* = 279) switched to denosumab, with a median of 8 days (IQR: 18) between doses.

**Conclusions:**

Significant gaps exist in the treatment of osteoporosis with denosumab and bisphosphonates in RAC placing residents at risk. Reasons for and interventions to address these gaps should be urgently explored, leveraging digital systems and RAC pharmacist services.

## Graphical abstract

This original research used medication administration data to explore adherence to treatment guidelines for the most common osteoporosis medications in long‐term residential aged care (RAC) in Australia. The paper highlights gaps in adherence to treatment guidelines that must be addressed to improve outcomes of older adults living in RAC.

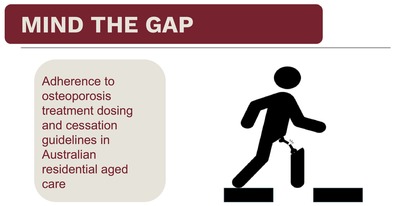

**What is already known about this subject**

- Use of denosumab for osteoporosis in RAC is increasing. Non‐adherence to 6‐monthly denosumab dosing and replacement with a bisphosphonate when ceased places residents at increased risk of spontaneous vertebral fractures.

**What this study adds**

- Using medication administration data, we explored adherence to denosumab treatment guidelines. Our data showed that 20% of residents on denosumab were exposed to dosing intervals longer than recommended and 98% of residents who ceased denosumab were not administered a bisphosphonate. These gaps in adherence to denosumab treatment guidelines need to be urgently addressed to improve care quality and outcomes of older adults living in RAC.

## 1 INTRODUCTION

In Australia, the total costs associated with osteopenia and osteoporosis are estimated to climb to $8.3 billion per year by 2033.^1^ Osteoporosis is common among residents in long‐term residential aged care (RAC), with bisphosphonates and denosumab the most frequent treatments.^2^ Bisphosphonates and denosumab reduce risk of fracture, mortality and associated health care costs.^3^, ^4^ Data from a sample of 9094 older people in 68 Australian RAC homes showed denosumab use increased between 2014 and 2017, whereas bisphosphonate use declined.^2^ The reduced pill burden and ease of administration of denosumab, a 6‐monthly injection compared with a weekly oral bisphosphonate tablet, may be one of the reasons for this change in use.

As noted in early clinical trials published in 2011, an important requirement of denosumab treatment is that doses are administered on a strict 6‐monthly schedule, as an increase in the dosing interval due to missed or delayed doses leads to a rapid increase in bone turnover and subsequent bone loss.^5^, ^6^ During this phase of high bone turnover, an increased risk of spontaneous vertebral fractures has been reported.^5^, ^6^ A 2017 post hoc analysis of the FREEDOM trial, examining denosumab discontinuation, recommended the need for rapid transition to alternative antiresorptive treatment after denosumab discontinuation due to the increased vertebral fracture risk.^6^ These recommendations are reflected in treatment guidelines, which recommend that if denosumab is ceased, bisphosphonates be used for 12 months (see Table 1).^4^, ^7^, ^8^, ^9^ The rapid bone loss experienced after denosumab discontinuation is in contrast to treatment with bisphosphonates that have a persistent effect after discontinuation. Thus, for those who respond well to 5 years of treatment with a bisphosphonate and who are not at high risk of vertebral fracture, a ‘drug holiday’ or treatment break of up to 5 years can be considered.^10^ Guidelines on cessation of and switching between bisphosphonates and denosumab are summarized in Table 1.

**TABLE 1:** A summary of treatment guidelines for denosumab and bisphosphonates.

	Therapeutic guidelines^7^	RACGP guidelines^4^	International guidelines (UK NOGG & NICE, European Society of Endocrinology)^8^, ^9^
Denosumab dosing schedule	To be administered every 6 months	To be administered every 6 months	To be administered every 6 months ±4 weeks
Cessation of denosumab	To be continued indefinitely or replaced with bisphosphonate. A suggested regimen, based on limited clinical trial data, is to start oral alendronate no more than 6 months after the last dose of denosumab and continue for 12–24 months.	Denosumab therapy should not be interrupted. If ceased, transition to bisphosphonate therapy for a minimum of 12 months. An oral bisphosphonate should be commenced within 4 weeks of the missed denosumab dose.	Denosumab should not be delayed or stopped without subsequent antiresorptive. Consider giving bisphosphonates and then stopping for a drug holiday.
Switching from a bisphosphonate to denosumab	No guidelines	No guidelines	If high‐risk, continue with bisphosphonate or switch to denosumab. There is a lack of evidence to guide such decisions.
Cessation of bisphosphonates	Consider stopping after 5 years (oral) or 3 years (IV). Continue for up to 10 years if at high‐risk of fracture. Re‐check BMD 2–3 years after stopping.	Reconsider the need to continue bisphosphonate therapy after 5–10 years in postmenopausal women and men over the age of 50 years who have responded well to treatment (T‐score ≥−2.5 and no recent fractures).	Initial treatment period of 5 years, with continuation if at high‐risk of fracture.

Abbreviations: BMD: bone mineral density; IV: intravenous; NICE: National Institute for Health and Care Excellence; NOGG: National Osteoporosis Guideline Group; RACGP: Royal Australian College of General Practitioners; UK: United Kingdom.

An analysis of Australian general practice data (MedicineInsight) from more than 3300 GPs in 705 general practices between 2012 and 2017, identified low rates of bisphosphonate use after denosumab cessation prompting calls for interventions to address this gap.^11^ Currently, little is known about adherence to the 6‐monthly denosumab dosing schedule and cessation guidelines in RAC, nor about practices of switching from a bisphosphonate to denosumab. A recent analysis of 5700 medication incidents in RAC showed that delays and omissions in medication dosing and supply account for a substantial portion of incidents.^12^ These medication management challenges in RAC place residents receiving denosumab at a heightened risk of adverse outcomes. Thus, we aimed to examine adherence to denosumab dosing and cessation guidelines, practices of ceasing or switching bisphosphonate treatment and how these vary in metropolitan *vs*. regional areas in Australian RAC using routinely collected medication administration data.

## 2 METHODS

### 2.1 Study setting

We conducted a retrospective cohort study using medication administration records from a sample of 461 RAC homes across Australia. The study period was from 1 January 2018 to 31 December 2022. The research was approved by the Macquarie University Human Research Ethics Committee (ID: 12031). This study is reported in accordance with the REporting of studies Conducted using Observational Routinely collected health Data (RECORD) statement.^13^

### 2.2 Participants

**FIGURE 1:**
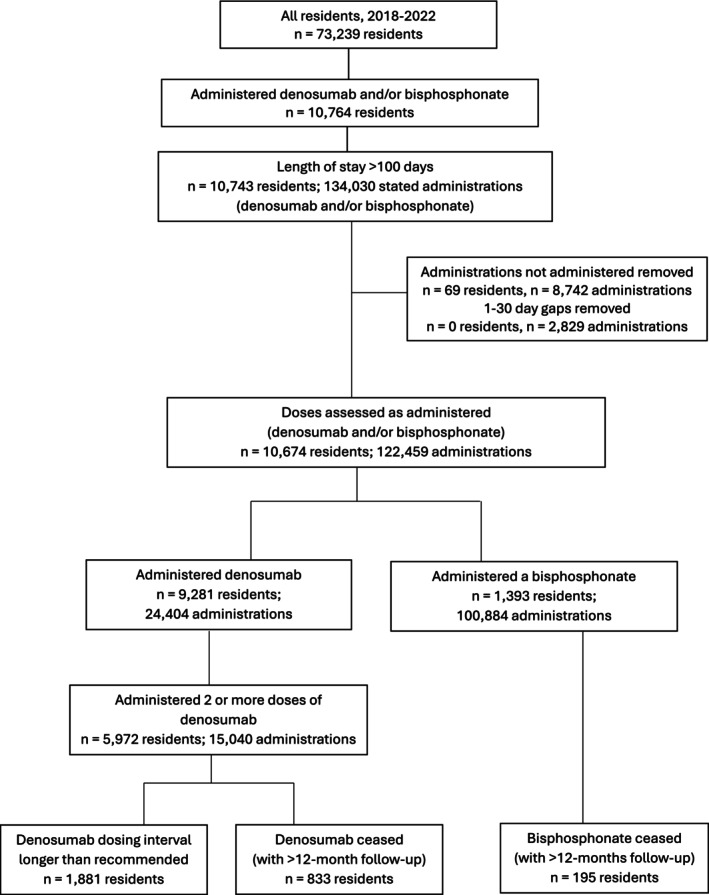
Study inclusion flow chart.

We included residents who had resided in RAC for more than 100 days and were administered denosumab and/or a bisphosphonate during their stay. No residents were administered the newer agents for osteoporosis, teriparatide (registered with Therapeutic Goods Association [TGA] in 2003) or romosozumab (registered with TGA in 2021). For the analysis examining denosumab or bisphosphonate cessation, we only included residents who were present in the homes for a minimum of 12 months after cessation. An individual was considered to be present in a facility when administered one or more medications during the study period. Figure 1 shows the study inclusion flow chart.

### 2.3 Data source

De‐identified medication administration data were extracted for homes that used the same electronic medication administration system, BESTMED. Data contained residents' demographics (e.g., age and gender), health condition (e.g., dementia, chronic respiratory condition, Parkinson's disease or diabetes), facility location and daily medication administration records. Health conditions were recorded as free‐text entries, which were processed using a previously developed health macro to identify specific conditions.^14^ Daily medication administration details comprised medication names, administration times and reason codes for any missed administration. We coded medications using the World Health Organization's Anatomical Therapeutic Classification (ATC). The individual medications and their corresponding ATC codes included in the analyses are shown in Table 2. Denosumab 120‐mg preparations were excluded as they are indicated for bone tumour or metastases with a four‐weekly dose.

**TABLE 2:** WHO ATC^a^ codes and generic names included in analyses.

ATC code	Drug name	Classification
M05BX04^a^	Denosumab	Denosumab
M05BA04	Alendronate	Bisphosphonate
M05BA07	Risedronate	Bisphosphonate
M05BA08	Zoledronate	Bisphosphonate
M05BB02	Risedronate & calcium	Bisphosphonate
M05BB03	Alendronate & calcium	Bisphosphonate
M05BB04	Risedronate & calcium & colecalciferol	Bisphosphonate
M05BB05	Alendronate & calcium & colecalciferol	Bisphosphonate
M05BB07	Risedronate & colecalciferol	Bisphosphonate
M05BB08	Zoledronate & calcium & colecalciferol	Bisphosphonate

*Note*: Denosumab 120‐mg preparations were excluded as they are indicated for bone tumour or metastases with a 4‐weekly dose.

^a^ World Health Organization's Anatomical Therapeutic Classification.

### 2.4 Statistical analysis

All data preparation and analyses were performed in Stata (version 18.5).^15^

To examine adherence to denosumab dosing interval guidelines, we examined the number of days between consecutive denosumab doses using recorded administration dates. We excluded administration records where a reason for a non‐administered dose was entered, such as ‘person absent’, ‘no stock available’, ‘person in hospital’ or ‘dose omitted’. Reasons for non‐administration were only recorded in the electronic record from 2022. Non‐administration records often appeared daily over extended periods for a small number of residents. Thus, we excluded denosumab doses recorded within 1–30 days of each other, as these likely reflected non‐administered or missed doses (refer to Appendix A for examples). The number of days between denosumab administrations was categorized as too few (31–150 days), appropriate (151–210 days) and too many (211 days and over). We calculated the percentage of denosumab dosing intervals >210 days and the percentage of residents affected by longer than recommended dosing intervals.^7^, ^8^, ^9^

To examine adherence to denosumab cessation guidelines and bisphosphonate cessation practices, we only included residents for whom there was at least a 12‐month follow‐up available. For these residents, we identified those who ceased denosumab without bisphosphonate replacement during the observation period after the last denosumab dose.^7^, ^8^, ^9^ We also identified residents who ceased a bisphosphonate and calculated the percentage who commenced denosumab and the number of days between the last bisphosphonate dose and the first denosumab dose.

The number of days between administration was summarized using descriptive statistics such as means, medians and inter‐quartile ranges (IQR). A chi‐squared test of independence was conducted to examine the relationship between geographic location (metropolitan *vs*. regional areas as defined by the Australian Bureau of Statistics) and non‐adherence to denosumab dosing and cessation guidelines.

### 2.5 Nomenclature of targets and ligands

Key protein targets and ligands in this article are hyperlinked to corresponding entries in https://www.guidetopharmacology.org/ and are permanently archived in the Concise Guide to PHARMACOLOGY 2021/22.^16^

## 3 RESULTS

**FIGURE 2:**
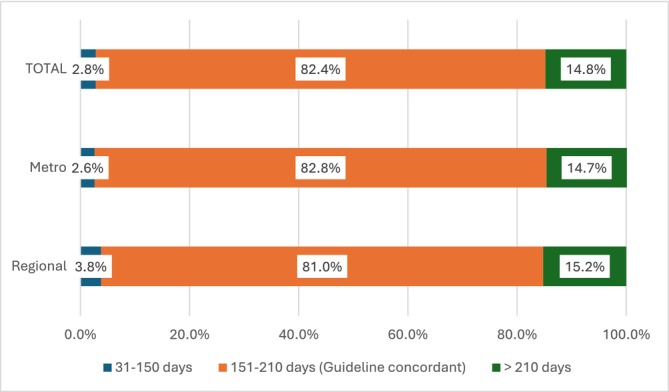
Number of dosing intervals concordant with guidelines for denosumab administrations by regionality (*n* = 9281 residents).

Of the 73 239 residents from 461 RAC homes, 10 674 residents in 413 homes met the inclusion criteria and were administered denosumab or a bisphosphonate between 2018 and 2022 (Figure 2).

The characteristics of the included residents are shown in Table 3. The median age of residents was 90 years, 71.5% were female, 75.7% lived in metropolitan areas and 44.7% had a recorded diagnosis of dementia. More residents (*n* = 9281; 86.9%) were administered denosumab than a bisphosphonate (*n* = 1393; 13.1%), and only 3.0% (*n* = 319) were administered both medications during the study period. The mean number of denosumab doses administered was 2.3 (SD = 1.9) per resident.

**TABLE 3:** Characteristics of residents administered a bisphosphonate or denosumab, 2018–2022 (*n* = 10 674).

Characteristics	
Gender, *n* (%)	
Female	7632 (71.5%)
Male	1892 (17.7%)
Other^a^	1150 (10.8%)
Age, median (IQR)	90 (85.0, 95.0)
Length of stay, median (IQR)	589 (IQR 334–943)
Number of administrations per resident (of any osteoporosis med), median (IQR)	3 (IQR 1–5)
Health conditions, *n* (%)	
Diabetes	1930 (18.1%)
Chronic respiratory conditions	3165 (29.7%)
Parkinson's disease	1074 (10.1%)
Dementia	4766 (44.7%)
Regionality, *n* (%)	
Metropolitan areas	8805 (75.7%)
Regional areas	2589 (24.3%)
State, *n* (%)	
New South Wales	5189 (48.6%)
Victoria	2572 (24.1%)
Queensland	1445 (13.5%)
Other^b^	1468 (13.8%)
Administered at least one dose of denosumab, *n* (%)	9341(87.5%)
Administered at least one dose of a bisphosphonate, *n* (%)	1393 (13.1%)
Risedronate	805 (7.5%)
Alendronate	567 (5.3%)
Zoledronate	21 (0.2%)

^a^ Includes missing values.

^b^ Includes the Australian Capital Territory, South Australia, Tasmania and Western Australia.

### 3.1 Adherence to 6‐monthly denosumab administration schedule

Of the 9281 residents who were administered denosumab, 5972 were administered more than one dose. In total, there 15 040 dosing intervals between consecutive denosumab administrations. Of these intervals, 82.4% (*n* = 12 394/15 040) were concordant with guidelines (151–210 days between doses) and 14.8% (*n* = 2222) were longer than recommended by guidelines (≥210 days), with a maximum interval of 1441 days (median 180 days, IQR: 3). In metropolitan and regional areas, 14.7% (*n* = 1750) and 15.2% (*n* = 472) of denosumab dosing intervals were longer than recommended, respectively (*χ* ^2^ = 3.5, *p* < .01; Figure 2).

Of all residents on denosumab, 20.3% (*n* = 1881) had a dosing interval longer than recommended, and 3.2% (*n* = 279) residents had 2 or more longer than recommended dosing intervals.

### 3.2 Adherence to denosumab cessation guidelines

A total of 847 residents ceased denosumab during the study period and were observed for at least 12 months post‐cessation. Of these residents, and contrary to guidelines, 98.2% (*n* = 833) ceased denosumab without bisphosphonate replacement. There were 14 residents who ceased denosumab and had one or more subsequent bisphosphonate administrations, with a median of 8 days from the last denosumab dose to the first bisphosphonate administration (IQR: 18). In metropolitan and regional areas, 98.9% (*n* = 603/610) and 97.0% (*n* = 230/237) of residents ceasing denosumab did so without bisphosphonate replacement, respectively (*χ* ^2^ = 3.6, *p* < .001).

### 3.3 Cessation of bisphosphonates

There were 474 residents who ceased a bisphosphonate with a minimum of 12 months follow‐up. Of these residents, 58.9% (*n* = 279) switched from a bisphosphonate to denosumab. The median dosing interval between the last bisphosphonate dose and the first denosumab dose was 8 days (IQR: 18). Of these 474 residents, 41.1% (*n* = 195) ceased a bisphosphonate with no commencement of denosumab.

## 4 DISCUSSION

Our study has demonstrated that a significant portion of aged care residents are at risk of harm due to non‐adherence to denosumab dosing and cessation guidelines. Of those administered denosumab during the study period, 20% had dosing intervals longer than recommended. Of the residents who ceased denosumab, 98.2% (*n* = 833) had no bisphosphonate replacement. Non‐adherence to the dosing and cessation guidelines for denosumab places residents at high risk of adverse events such as spontaneous vertebral fractures,^6^ fractures from falls and hypocalcaemia in renal impairment. In RAC populations, osteoporosis is common^2^ and falls and related injuries are a significant contributor to poor health outcomes and wellbeing. Thus, appropriate management of osteoporosis is critical to supporting the quality of life of older adults living in RAC.^17^

Consistent with other literature, we have demonstrated that greater attention to osteoporosis treatment among this high fracture‐risk population is warranted. Studies in community‐dwelling populations have also highlighted a lack of adherence to the 6‐monthly dosage schedule for denosumab. A study of 148 554 community‐dwelling patients in Canada found that approximately one quarter did not receive denosumab administrations within the 6‐month recommended window, during and after the COVID pandemic.^18^ A European study of 1500 patients receiving denosumab in routine practice, mainly prescribed by specialists, in Germany, Austria, Greece and Belgium, reported that 10% did not adhere to the 6‐month denosumab dosing schedule.^19^ Similarly, a study of 768 patients initiated denosumab in a hospital in Singapore showed adherence to denosumab was significantly lower during COVID‐19, at 63.9%, than in the pre‐COVID‐19 period at 75.4%.^20^ In a specialty community private practice in the United States, of 1158 patients, 64% adhered to the 6‐monthly denosumab dosing schedule.^21^ A smaller study in Ireland of 98 patients in a geriatric rehabilitation unit found 57% had one denosumab injection with no subsequent doses and no replacement therapy with a bisphosphonate.^22^ To the best of our knowledge, our study is the first to examine this issue in RAC, where the population is at higher risk of adverse events as a result of non‐adherence due to multimorbidity and a higher risk of falls.^23^

We found that only a small proportion (1.7%) of residents were administered a bisphosphonate after ceasing denosumab. This may be due in part to the fact that the Royal Australian College of General Practice (RACGP) osteoporosis guidelines included the recommendation of bisphosphonate replacement in 2024.^4^ The majority of the denosumab cessations in our study occurred between 2021 and 2022 (65.3%) before the update to the RACGP guidelines. However, importantly, earlier trials and guidelines recognized rapid bone resorption with delayed denosumab doses^5^, ^24^ and landmark trials recommended the use of antiresorptive therapy after denosumab cessation in papers as early as 2017.^6^, ^25^ International guidelines were updated to reflect these recommendations in 2019.^9^ It is also important to acknowledge other possible reasons for the non‐use of bisphosphonates after denosumab cessations. These may include renal impairment, which is a contraindication for bisphosphonates, with chronic kidney disease affecting an estimated 42% of older adults aged over 75 years.^26^ The inability to meet oral bisphosphonate dosing requirements (e.g., staying upright for 30 min) and gastroesophageal irritation may also limit bisphosphonate use after denosumab cessation. End‐of‐life care may also be a reason for cessation of denosumab without bisphosphonate replacement. However, our analysis examined whether bisphosphonates were administered in residents with a minimum 12‐month follow‐up after denosumab cessation, to account for end‐of‐life care. Further, even during palliative care, continuation of denosumab is recommended as the net benefit would outweigh the risk of potential harm such as pain from rebound vertebral fracture.^27^ Despite the possible clinical reasons, the low rate of adherence to bisphosphonate replacement is of concern in this population and warrants further investigation.

System‐level interventions to support adherence to denosumab dosing and cessation guidelines are needed. A recent review of pharmacotherapy for osteoporosis outlined the importance of enhanced adherence strategies.^28^ Several strategies have been found effective in improving adherence to denosumab treatment in the community setting. In Ireland, free access to GP services for follow‐up appointments to check adherence may have resulted in better medication persistence.^22^ In Singapore, the odds of adherence to denosumab dosing were higher if patients were managed by an endocrinologist.^20^ Models involving pharmacists with responsibility for adherence to treatment regimens were found to bring about improvement in China.^29^ This included dissemination of patient information at the initial dose and a reminder text 1 week before the next dose, with contact made with the patient if this dose was not given.^29^

Although many of the interventions described in prior studies focus on supporting patient adherence, they may be adapted to the RAC setting. In the Australian RAC, there are several opportunities for pharmacists to support denosumab dosing and cessation guideline adherence. Firstly, the pharmacy supplying medicines to RAC can ensure stock availability for timely dosing, and follow‐up with GPs regarding bisphosphonate replacement when denosumab is ceased. Secondly, RAC homes have access to clinical pharmacy services that can support monitoring of adherence to osteoporosis treatment guidelines, as well as provide education for prescribers and carers.^30^, ^31^

The rapid uptake of electronic medication management systems in Australian RAC also provides opportunities for digital prompts and workflows to support adherence to treatment guidelines. Electronic medication systems include prescribing, administration and dispensing functionality and are used by pharmacists, RAC staff and GPs and other prescribers. These systems could be enhanced with the addition of clinical decision support specific to adherence to denosumab dosing intervals and guidelines for switching between denosumab and bisphosphonates. Systems should also support documentation and resolution of delays in administration if there is no stock available at the RAC. Ensuring there are mechanisms for staff to record the reasons that doses were not administered on the electronic medication chart is critical to care documentation and should be a feature of systems to allow improved supply mechanisms and monitoring of doses. Finally, reporting of when doses of denosumab are due could facilitate ordering from suppliers in advance to avoid delays in administration.

In our cohort, we found that 41% of residents who ceased a bisphosphonate had a drug holiday for at least 12 months. However, the majority of residents who ceased a bisphosphonate commenced denosumab. In this group of residents, the time between the last bisphosphonate dose and commencing denosumab varied widely, from 1 to 1039 days. The variation may reflect a drug holiday with monitoring of bone mineral density. However, it should also be noted that guidelines do not provide an optimal dosing interval when switching from a bisphosphonate to denosumab,^4^, ^7^, ^8^, ^9^ which likely contributes to the variation in practice. This limitation in current guidelines should be addressed.

Our analysis has several strengths, including the longitudinal design, use of medication administration data and data from a large number of residents and RAC homes. However, although our study aimed to quantify the extent of guideline adherence, our dataset did not contain details of the reasons for the longer‐than‐recommended denosumab dosing intervals and the lack of bisphosphonate replacement after denosumab cessation. This limited us from assessing the appropriateness of these gaps. There is the possibility that for a small number of residents, denosumab or zoledronate infusion was administered in a specialist's room in the community or in hospital and thus was not recorded on the RAC home electronic medication administration chart, which would not be captured in our data. In our experience in this setting, it is likely that this would represent a small proportion of residents. Nonetheless, the low rates of bisphosphonate replacement and frequent longer than recommended dosing intervals warrant concern in this population.

## 5 CONCLUSION

There are critical gaps in the adherence to denosumab dosing and cessation guidelines in Australian RAC, placing residents at risk of harm. Interventions to address this are urgently needed and should leverage digital systems and pharmacist services to improve prescribing, supply and medication administration, to ensure safe use of these medicines and better outcomes for older adults in RAC.

## AUTHOR CONTRIBUTIONS

Marea O’Donnell, MD Bayzidur Rahman, Magdalena Z Raban, Nasir Wabe, Joahanna I Wetbrook and Isabelle Meulenbroeks contributed to the study conception and design. Material preparation, data collection and analysis were performed by Marea O’Donnell, MD Bayzidur Rahman, Magdalena Z Raban and Isabelle Meulenbroeks. The first draft of the manuscript was written by Marea O’Donnell and Ian D Cameron and Mark Morgan provided input from a clinical perspective. All authors commented on previous versions of the manuscript. All authors read and approved the final manuscript.

## CONFLICT OF INTEREST STATEMENT

M.R. and J.I.W. have received grant funding from the Australian Commission on Safety and Quality in Health Care. J.I.W. has received research funding from the NHMR and MRFF. J.I.W. holds an NHMRC Leadership Investigator Grant. J.I.W. receives fees for teaching for Harvard University, as a Co‐Director of the Safety, Quality, Informatics & Leadership Program. M.M. receives payment for consultancy services from RACGP, PHNs, DoHAC and Australian Health Policy Collaboration of Victoria University.

## Data Availability

The de‐identified data for this study were made available to the researchers under agreements that do not allow public sharing.

## APPENDIX A

Examples of denosumab administrations assessed as not administered

As the electronic medication system did not record delayed or non‐administered medication doses during the entire study period, we applied a rule of thumb to consecutive administrations of denosumab to identify administrations vs delayed doses. Doses of denosumab administered 1–30 days after a previous denosumab dose were assessed as not administered and excluded from the analysis, as shown in the figure above and described below.

Resident 1:

Denosumab administered upon admission then 180 days later as expected given denosumab is indicated to be given every 6 months. Both administrations were included in the analysis.

Resident 2:

Initial consecutive daily administrations (1 day gaps) are omitted, with the final one included as administered. A second administration at 190 days is included as administered, with subsequent administrations of <30 days omitted.

Resident 3:

Initial consecutive administrations with 1‐day gaps are omitted, with only the final one included. Then, the next two administrations that are 34 days apart are included, as cut off for admission was 30 days. After second dose, administrations 1–2 days apart are recorded but are omitted as not administered. Next dose is recorded 190 days after the second dose.
